# Questioning Domain Adaptation in Myoelectric Hand Prostheses Control: An Inter- and Intra-Subject Study

**DOI:** 10.3390/s21227500

**Published:** 2021-11-11

**Authors:** Giulio Marano, Cristina Brambilla, Robert Mihai Mira, Alessandro Scano, Henning Müller, Manfredo Atzori

**Affiliations:** 1 Information Systems Institute, University of Applied Sciences Western Switzerland (HES-SO Valais), 3960 Sierre, Switzerland; giulio.marano92@gmail.com (G.M.); manfredo.atzori@hevs.ch (M.A.); 2 Department of Computer, Control, and Management Engineering, La Sapienza University, 00185 Rome, Italy; 3 UOS STIIMA Lecco-Human-Centered, Smart & Safe, Living Environment, Italian National Research Council (CNR), 23900 Lecco, Italy; cristina.brambilla@stiima.cnr.it (C.B.); robertmihai.mira@stiima.cnr.it (R.M.M.); alessandro.scano@stiima.cnr.it (A.S.); 4 Department of Radiology, Medical Faculty, University of Geneva, 1211 Geneva, Switzerland; 5 Department of Neuroscience, University of Padua, 35122 Padua, Italy

**Keywords:** machine learning, EMG, biofeedback, transfer learning, random forest classifier

## Abstract

One major challenge limiting the use of dexterous robotic hand prostheses controlled via electromyography and pattern recognition relates to the important efforts required to train complex models from scratch. To overcome this problem, several studies in recent years proposed to use transfer learning, combining pre-trained models (obtained from prior subjects) with training sessions performed on a specific user. Although a few promising results were reported in the past, it was recently shown that the use of conventional transfer learning algorithms does not increase performance if proper hyperparameter optimization is performed on the standard approach that does not exploit transfer learning. The objective of this paper is to introduce novel analyses on this topic by using a random forest classifier without hyperparameter optimization and to extend them with experiments performed on data recorded from the same patient, but in different data acquisition sessions. Two domain adaptation techniques were tested on the random forest classifier, allowing us to conduct experiments on healthy subjects and amputees. Differently from several previous papers, our results show that there are no appreciable improvements in terms of accuracy, regardless of the transfer learning techniques tested. The lack of adaptive learning is also demonstrated for the first time in an intra-subject experimental setting when using as a source ten data acquisitions recorded from the same subject but on five different days.

## 1. Introduction

Amputation is one of the major reasons of disability [1]: it is estimated that 100.000 people have an upper limb amputation in the United States, and 57% of these are transradial amputees [2]. The principal causes of upper limb loss are traumatic events, followed by vascular diseases, congenic absence and cancer [3]. Upper limb amputation limits the daily life activity of a person heavily [4], although myoelectric prosthesis can restore the functionality of the hand using non-invasive EMG signal of the residual muscles [5]. The use of myoelectic signals has several advantages with respect to body-powered prostheses because the user does not need harnesses, the signal is recorded non-invasively on the skin and the effort required to control it is comparable to the one of an intact limb [6]. However, user acceptance is still low because of a lack of intuitive and dexterous control [7]: the rate of prosthesis abandonment is about 44% [8]. The control should be intuitive for the user, robust to arm and electrode positioning, adaptive to changes such as fatigue or sweating and easy to train [9].

In recent years, thanks to the advancement of robotics [10], control systems [11] and artificial intelligence [12], remarkable improvements have been made in the control of dexterous, robotic hand prostheses [13]. In particular, machine learning techniques allowed for developing surface electromyography (sEMG) prostheses that are capable to learn from each subject the myoelectric patterns corresponding to different hand movements [14]. However, such training procedures can be long (particularly to control a large number of hand movements), they are not robust to electrode re-positioning [1,9] (which can happen, for instance, after removing the prosthesis at night) and they can lead to considerable efforts for the patients. It was also found that inter-subject and inter-session variability are factors that may affect muscle coordination patterns [15]. Young et al. [16] found that a higher inter-electrode distance and a combination of longitudinal and transverse oriented channels can reduce the effects of electrode shift on the classification accuracy. The difficulties related to training myoelectric models increased the interest of scientific researchers for pre-built models [17]. Such models are expected to collect previous experience from several subjects and (through appropriate domain adaptation algorithms) they can be adapted to patients, accelerating model training. However, this approach can lead to divergence errors between different domains [18]. Over the years, several experiments lead to promising results in the domain of transfer learning [17,18,19,20].

One of the first studies about myoelectric signal divergence is from Castellini et al. [21]. They observed that myoelectric signals differ considerably between different subjects and that the use of pre-trained models should be bound to subjects who share a sufficient amount of similarities. This observation led to different approaches in order to take advantage of the prior knowledge of different subjects. Sensinger et al. [22] proposed several ways to concatenate source and target data in one model. Then, in order to improve non-adaptive baselines, Hypothesis Transfer Learning algorithms were employed in several studies. The advantage of these algorithms is that they do not need direct access to raw data exploiting models previously achieved from source subjects. Côté-Allard et al. [19] showed that transfer learning can lead to improved performance of hand gesture classifiers in three different datasets of able-bodied subjects using a convolutional network for the target domain, combining networks trained on the source with different activation functions. Kanoga et al. [23,24] acquired the same healthy subject for thirty consecutive days and applied domain adaptation on a linear discriminant analysis classifier, interpolating the mean vector and the covariance matrix of the calibration data of each day with the data recorded on the first day, and concluded that these methods allowed to adjust parameters for changes in positioning of the electrodes between different days. Other studies [25,26] applied transfer learning on convolutional neural networks (CNN) to improve model robustness on electrodes shifts: Ameri et al. [25] used a CNN model trained on data before shifting as a pretrained network and fine-tuned the model using few data of the same user after shifting; Wang et al. [26] transferred the parameters of the recurrent CNN model of the source domain to the EMG feature-extraction module of the target domain. Moreover, Liu et al. [27] applied domain adaptation techniques on a polynomial classifier, using the leave-one-out prediction error as a metric for the optimization algorithm, and on a linear discriminant analysis classifier with the Mahalanobis distance as a metric of consistency between prior models and the current training data. These algorithms were applied on both intact-limbed and transradial-amputee subjects for ten consecutive days, and it was found that the domain adaptation methods outperformed the baseline methods for both classifiers, especially with a small size of training data. Finally, Prahm et al. [28] applied domain adaptation exploiting the relationship between source and target domain: since data were recorded with an electrode grid, the distance between the electrodes was maintained equal even after the electrodes shifting, and therefore, they considered the shift on only one electrode and assumed linear feature changes between neighboring electrodes. They applied these approaches on both able-bodied subjects and transradial amputees and noticed that model performance increased with transfer learning on able-bodied subjects, but no relevant improvements were found in amputees.

Recently, transfer learning algorithms were used to train a model over the source domain to adapt it to a target domain with local adjustments of the tree parameters and its architecture [29].

Although different studies stated the efficacy of the use of domain adaptation on gesture recognition, they were usually applied on able-bodied subjects. Exploiting previously achieved results on intact subjects [30] from the Non-Invasive Adaptive Prosthetics (NinaPro) database [31], Gregori et al. [32] extended the study to amputee subjects and presented a novel framework for a realistic experimental setup. They found that, if the hyperparameters were properly tuned, transfer learning approaches showed the same performance of the standard methods that did not employ prior knowledge.

In this paper, we improve these results by applying two recent domain adaptation algorithms [29] to a random forest classifier (which is normally applied without hyperparameter optimization in the domain). A random forest classifier was already used on healthy subjects in combination with a regressor for discriminating reach to grasp strategies, obtaining good results [33]. These domain adaptation techniques modify the structure of decision trees within the forests generated by the source models in order to refine them on target repetitions. Our aims are: (1) to confirm and extend to a different data analysis workflow the results obtained in previous research; (2) to evaluate the quality of random forests as classifier for domain adaptation problems on sEMG data; (3) to extend the experiments to tests performed on data recorded from the same person but in data acquisition sessions of several days.

## 2. Materials and Methods

### 2.1. Domain Adaptation and Transfer Learning Algorithms

This section includes a step-by-step explanation of the models used in the domain adaptation and a description of the transfer learning algorithms used in our experiments.

#### 2.1.1. Source, Target and Test Sets

Given $S=[s_{1},s_{2},\ldots ,s_{n-1},s_{n}]$, where *n* is the number of subjects in our dataset, the first step is the signal feature extraction. We can define all signal features extracted as $D={\left\{x_{i},y_{i}\right\}}_{i=1}^{N}$, where $x_{i}\in R^{d}$ are the input samples, $y_{i}\in Y=\left\{1,\ldots ,G\right\}$ are the paired labels and G is the number of possible classes plus the rest pose. We can split our initial collection of subjects into a target model $S_{T}$ composed of a single subject and a source model $S_{S}$ with all remaining subjects.

In the next step, we divide the number of repetitions of $S_{T}$, using one part as a test $S_{T_{test}}$ and the remaining as intra-subject training in order to compare the results for domain adaptation. Then, $S_{S}$ is used as input to train a random forest classifier [34], which is a collection of decision trees. In this way, a forest trained on the source is obtained. In the same way, $S_{T_{train}}$ is passed to another random forest classifier, building another model trained on the target as result.

The domain adaptation step follows, where different algorithms transform the forest trained on the source and refine it on the target.

In conclusion, all previous forests are tested on $S_{T_{test}}$ and compared. This procedure is then repeated *k*-times, with k∈S, so that each subject takes the role of target at most once.

**Figure 1:**
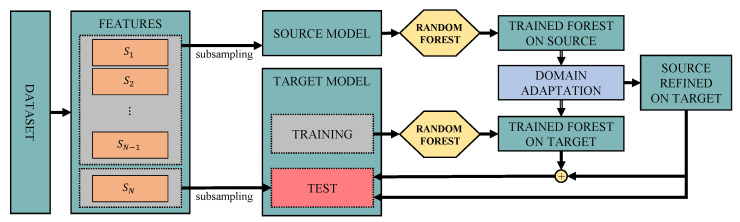
Block scheme for the domain adaptation model.

All steps described above are graphically explained in Figure 1.

#### 2.1.2. Domain Adaptation Algorithms

Given a number of sources, a domain adaptation algorithm builds a new classification model refining the source on target. We use three algorithms, all based on random forest classifiers. Structure Expansion/Reduction (SER) and Structure Transfer (STRUT) take forests trained with source model as input and adapt them to a new target domain [29].

Then, we define a MIX algorithm that uses ensembles from both SER and STRUT as input and mixes them. More specifically, we can describe domain adaptation algorithms as follows:

(1) *Structure Expansion/Reduction (SER):* Given a random forest $RF_{S}$ induced using the source data $S_{S}$, each decision tree (DT) is processed independently by the SER algorithm. First of all, the set $S_{v}^{T}$ of all labeled points in the target data $S_{T_{train}}$ that reach the node *v* is computed. Then, in the expansion phase, a full tree expands from each leaf *v* with respect to $S_{v}^{T}$. Lastly, with a bottom-up approach, the algorithm performs a reduction of the structure for each internal node *v*.
  This reduction is determined by two kinds of errors with respect to $S_{v}^{T}$:
  - Subtree error $E_{S}$;
  - Leaf error $E_{L}$.
  The subtree error is the empirical error of the subtree of which the root is *v*. The leaf error is defined to be the empirical error on *v* if it were to be pruned into a leaf. If the following condition holds:
  (1) $$E_{S}>E_{L}$$
  the subtree is pruned into a leaf node. The decision value at each leaf of the DT is obtained using the target (empirical) distribution. The SER algorithm then iterates these operations for each DT contained in the initial forest, building a new forest adapted on source.
(2) *Structure Transfer (STRUT):* While the SER algorithm acts on size of DTs inside $S_{S}$, the Structure Transfer algorithm changes the threshold. Since decision trees show similarity for similar problems, the STRUT algorithm exploits a top-down approach, adapting a DT trained on the source samples to the target samples by discarding all numeric threshold values in the tree. The values of the numeric thresholds are substituted by new thresholds τ(v) for a node *v* using the subset of target examples $S_{v}^{T}$ that reach *v*.
  If $S_{v}^{T}$ is empty for a node *v*, *v* is pruned because it cannot be reached in the target domain. At each leaf, the final decision value is computed on the target training data.
  To perform threshold selection for feature ϕ, STRUT uses two parameters:
  - Divergence gain *DG*;
  - Information gain *IG*.
  *DG* determines the distributional similarity, while *IG* quantifies the informative value of the threshold. The similarity is related only to those thresholds *x* whose *IG* is larger than the *IG* of any other $x^{\prime }$ in the ϵ-neighborhood of *x* for any sufficiently small ϵ>0. The STRUT algorithm searches for a threshold that gives a high similarity between the induced and the original distributions during the tree induction stage [35].
  The selection of the threshold can be considered as an optimization problem:
  (2) $$\begin{array}{cc} \max_{x} & DG(S_{v}^{T},\phi ,x,Q_{L},Q_{R})\\ s.t. & x\in R\\ \; & \forall x^{\prime }\in \left(x-\epsilon ,x+\epsilon \right)\;:\;IG\left(S_{v}^{T},\phi ,x\right)\geq IG\left(S_{v}^{T},\phi ,x\pm \epsilon \right) \end{array}$$
  where $Q_{L}$ and $Q_{R}$ are the left and right distribution, respectively.
(3) *MIX:* Once both SER and STRUT are applied, we obtain two distinct forests as a result. MIX is a combination of the two previous algorithms. This is a simple majority voting ensemble applied to all decision trees of both forests generated by STRUT and SER. As can be seen from the results, MIX does not simply average the results of the previous algorithms but often outperforms both of its constituents and thus is the second best solution. An intuitive explanation described in [29] about this result is given in Results.

### 2.2. Experimental Setup

The experimental setup is based on the “realistic setting” proposed in a previous work [32]. The setting was considered as “realistic” as it exploits real data coming from the Ninapro Dataset, recorded during the execution of daily life gestures. Section 2.2.1 presents the data used in all experiments. Then, the general settings and details about the experiments are described in Section 2.2.2.

#### 2.2.1. Data

**Figure 2:**
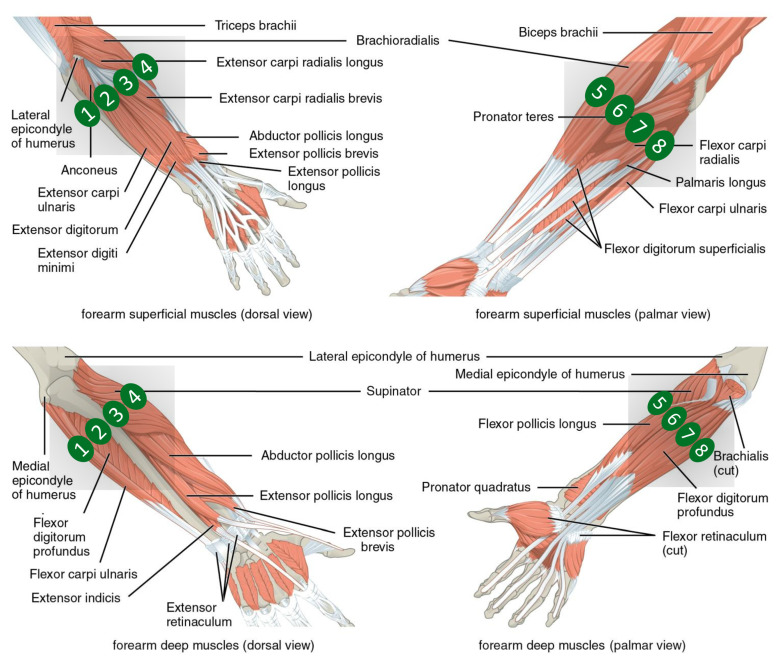
Positioning of the electrodes and underlying specific muscles. The image was adapted using file licensed under the Creative Commons Attribution 4.0 International license (Picture was adapted using https://upload.wikimedia.org/wikipedia/commons/7/73/1120_Muscles_that_Move_the_Forearm.jpg (accessed on 1 November 2021) from the Textbook OpenStax Anatomy and Physiology (source: https://cnx.org/contents/FPtK1zmh@8.25:fEI3C8Ot@10/Preface, accessed on 1 November 2021)).

The data used in this work are from the NinaPro database (http://ninapro.hevs.ch/, accessed on 1 November 2021) [31,36], one of the largest publicly available databases that contains sEMG data of a wide range of distal upper limb movements. We use three NinaPro datasets for the experiments (namely NinaPro DB2, DB3 and DB6). As reported in Table 1, in all three cases, the acquisitions were made with Delsys Trigno sEMG sensors. This choice was made at the moment of data acquisition in order to allow the combination of the different datasets in future studies. The following subsections provide a brief presentation of the datasets, which are described in more details in the reference papers [31,36]. An illustration of the available EMG channels in this dataset is presented in Figure 2.

(1) *NinaPro DB2 and DB3-Single acquisitions of intact subjects and transradial amputees:* The two datasets include 40 intact subjects and 11 amputees. Three amputees $\left[s_{1},s_{6},s_{7}\right]$ are excluded because their data acquisitions are not complete. Each subject executed 40 movements 6 times. Each movement repetition lasted 5 s and was followed by 3 s of rest. Twelve Delsys Trigno Wireless electrodes were used to record the sEMG data from the forearm of the subjects. Following a recently employed approach [7], a sliding window of 200 ms and an increment of 10 ms were used for the extraction of signal features. Therefore, the time windows were split into train and test sets for the classifiers, considering repetitions (1,3,4,6) for the training and repetitions 2 and 5 for test. A factor of 10 at regular intervals was used to subsample the training set in order to reduce the computational demands.
(2) *DB6-Intact subjects acquired for 5 consecutive days:* This dataset is composed of 10 intact subjects and targets the analysis of data acquisition repeatability in the same subjects. Each subject executed 7 hand grasps 12 times, two times per day, for 5 consecutive days. Each grasp is followed with a few seconds of rest. During the acquisition of the movements, fourteen electrodes recorded sEMG data. Eight electrodes are positioned as the first eight electrodes in NinaPro DB2 and DB3 (i.e., equally spaced around the forearm at the height of the radio-humeral joint). The windowing procedure follows the same approach described for the previous datasets. For each session, repetitions (1,3,4,6,7,9,10,12) were dedicated to training, while repetitions (2,5,8,11) were used as test. In this case, the training set was also subsampled by a factor of 10 at regular intervals.

The standardized data were used according to the protocol already proposed for control by Englehart and Hudgins [7], where features were extracted from a sliding window of 200 ms and an increment of 10 ms. As described in the papers presenting the datasets, sEMG signals were filtered from 50 Hz (and harmonics) power-line interference using a Hampel filter [31,36]. The resulting set of windows was subsequently split in the training set and test set as inputs for the classifier [32]. The sEMG representation used in this setting was the average of the marginal discrete wavelet transform (mDWT), mean absolute value (MAV) and variance (VAR) features [37].

#### 2.2.2. Experiment Settings

One of the novelties of this paper is the use of a random forest classifier for domain adaptation on sEMG data. It was suggested that the incorrect optimization of hyperparameters was the main cause of transfer learning and domain adaptation improvements presented in previous literature [32]. In fact, classifiers such as the SVM need a grid search to find the best hyperparameters. Using random forests makes the optimization phase easier. The number of 100 trees was fixed for each forest and for all the experiments. This setup has shown high level performance (comparable to SVM) in previous results on sEMG data [31]. This approach was used for both the forests generated for the construction of the target model and for the source model.

The same number of trees was also used for the domain adaptation algorithms SER and STRUT.

The following eight classification performances are compared in each experiment:

- Source only;
- Target only;
- SER;
- STRUT;
- MIX;
- Voting ensemble (SER, Target Only);
- Voting ensemble (STRUT, Target Only);
- Voting ensemble (MIX, Target Only).

**Figure 3:**
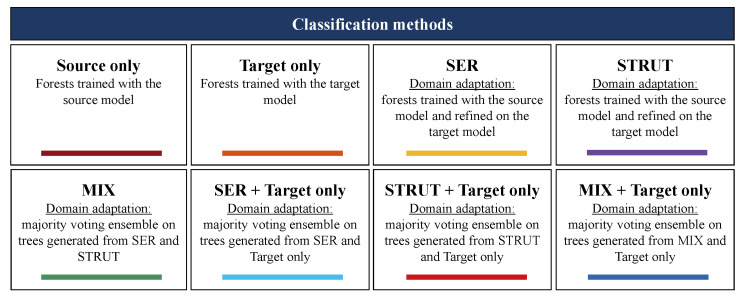
Scheme of the classification methods employed in each experiment.

While the first five values are explained in the previous sections, the last three follow the same observation as for the MIX algorithm, with the aim of exceeding the accuracy obtained by the two individual components separately. Each of them represents a separate voting ensemble of which the underlying model is the union of all decision trees inside $S_{T_{train}}$ and each algorithm presented in Section 2.1.2 independently. The methods are summarized in Figure 3.

The experiments on DB2, DB3 and DB6 are conducted as follows.

(1) *DB2 and DB3:* The first experiments replicate the experiments previously cited:
  - Intact–Intact: the classification of each subject from DB2 exploits prior knowledge of remaining subjects of DB2.
  - Amputee–Intact: the classification of each subject from DB3 exploits prior knowledge of remaining subjects of DB3 plus all subject of DB2.
  - Amputee–Amputee: the classification of each subject from DB3 exploits prior knowledge of remaining subjects of DB3.
  In the training set, the subsets from 1 to 4 repetitions were taken into account for training. In each case, the k-fold cross validation was used for the optimization of the target model, with each fold corresponding to samples of one repetition. The source models, instead, were trained using all repetitions.
(2) *DB6:* Due to the very high number of repetitions for each subject (120), two simplified experimental settings were chosen:
  - Intra-subject: each subject of DB6 exploits prior knowledge of the remaining repetitions of the same subject.
  - Inter-subject: each subject of DB6 exploits prior knowledge of the remaining repetitions of the same subject plus all remaining subjects of DB6.

For both experiments, the target model is composed of 12 repetitions of the fifth afternoon, while the remaining repetitions of each subject are used to build the source model. In the intra-subject setup, we considered almost all possible subsets including 1–8 repetitions. In each case, the target model was optimized using k-fold cross validation, with each fold corresponding to samples of one repetition. In the inter-subject setup, the target model was trained using all 8 repetitions of the same session only. In both cases, the source model was optimized using a k-fold cross validation, where k is the number of repetitions from other sessions of the same subject used as target, plus (for the inter-subject setup) the total number of repetitions of each other subject.

## 3. Results

**Figure 4:**
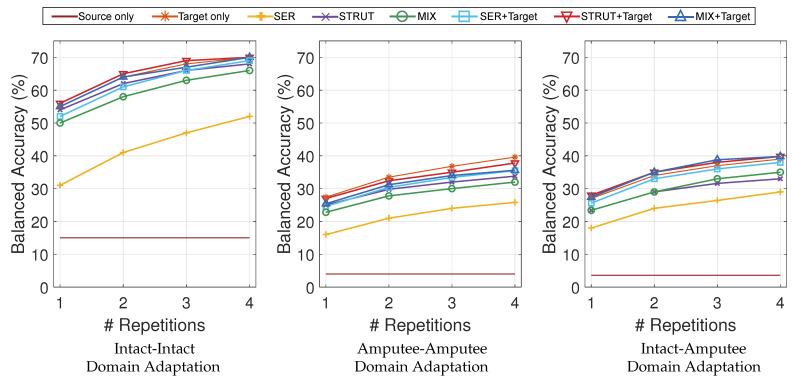
DB2 and DB3 Results: inter-subject balanced classification accuracy as a function of number of training repetitions on target.

**Figure 5:**
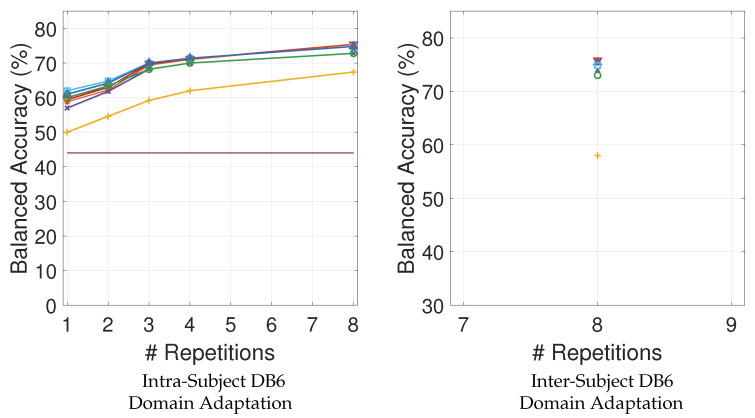
DB6 Results: intra-subject and inter-subject balanced classification accuracy as a function of number of training repetitions on target.

In Figure 4 and Figure 5, the balanced classification accuracy is reported as a function of the number of training repetitions on the target. Domain adaptation does not improve movement classification accuracy in comparison to no-transfer learning, neither when pre-training is performed on different subjects, nor when pre-training is performed on different acquisitions of the same subject.

In Figure 4, the first set of experiments, performed on NinaPro DB2 and DB3, extends results obtained previously on SVMs. Results are reported in details in Table 2. Using random forests domain adaptation for these experiments offers a perspective of the problem that is influenced by less variables, since the classifier is normally applied without hyperparameter optimization procedures in the domain. Such procedures had been recently presented as a possible source of errors for domain adaptation works based on SVMs [32].

The SER algorithm has a performance that is lower than the STRUT algorithm, while the latter almost perfectly overlaps with the target-only result. Given the low performances of the SER algorithm, it is not surprising that the MIX algorithm does not give the best results. The plots also include the source model tested directly on the target (the flat series of data with the lowest performance in the plot). This result highlights (especially for amputees) how much the high variability between different subjects affects classification performance. Indeed, the classification accuracy is lower when amputee subjects are included, and it is higher when only intact subjects are considered.

A further novel result is that domain adaptation does not improve movement classification accuracy even when the data come from the same subject. The domain adaptation experiments using several data acquisitions recorded from the same person in different moments show that the “target only” model almost constantly provides results in line with the ones obtained by the domain adaptation models (Figure 5). The voting ensemble between target repetitions combined with the STRUT algorithm obtains a small improvement of the classification accuracy when one or two repetitions are considered in Figure 5 (left).

Finally, domain adaptation from other subjects does not improve classification accuracy even when pre-training on several data acquisitions from different subjects. It is not possible to notice any improvement in Figure 5 (right), showing that the addition of information is ineffective even when relying on such a high number of repetitions of the source. The results are also portrayed in Table 3.

## 4. Discussion

The results show that inter-subject domain adaptation does not improve classification accuracy, and it extends the result to intra-subject models computed from different acquisitions of the same subject. This result confirms and extends previous results [32] and is in partial disagreement with several previous works on domain adaptation.

While previously this conclusion was explained in relationship to SVM parameter optimization, in our case the result is obtained using a random forest classifier (with a fixed configuration) and several new transfer learning methods. The domain adaptation methods tested in this study performed as well as the target-only baseline, which did not consider the source information. Similarly to previous findings [29], the STRUT algorithm gives better results with respect to the SER when the correspondence between features is maintained, while the SER algorithm outperforms STRUT on inverse problems. From our results, the MIX algorithm obtained a performance that was closer to the best performance, demonstrating that it had not the average performance of the SER and STRUT. SER and STRUT algorithms act differently on the same tree: the SER algorithm changes the size of the original tree, adding depth in the expansion phase and reducing the size of branches in the reduction one; the STRUT algorithm, instead, maintains the original size of the tree, modifying the thresholds. Therefore, the MIX forest turns out to be a more diverse forest, in which the pairwise correlation between two trees, derived from the same original tree, is low.

Our classification accuracy results are lower than some of the results previously proposed [32], but this is probably due to differences in the metrics used. While we preferred to use balanced classification accuracy (due to the unbalanced multi-class nature of the classification problem), unbalanced classification accuracy was most likely used for the realistic setting in the previous work. The difference in accuracy can thus be explained considering the high incidence of the rest in the dataset (which is classified with high accuracy).

Another interesting result is introduced by the experiments performed on the data from the same subject. The intra-subject experiment shows that using the previous experience of the same subject, there is basically no improvement compared to the case of no transfer learning. The most intuitive explanation for this result (also reported by Palermo et al. [36]) is that the re-positioning of the electrodes at each session produces substantially different results, even for the same subject.

The results from this work may impact real-life settings for people with hand prostheses. In fact, a major challenge limiting the use of dexterous robotic hand prostheses controlled via electromyography and pattern recognition relates to the important efforts required to train complex models from scratch. To overcome this problem, several studies in recent years proposed to use transfer learning, combining pre-trained models (obtained from prior subjects) with training sessions performed on a specific user. Differently from several previous papers, our results show that there are no appreciable improvements in terms of accuracy, regardless of the transfer learning techniques employed. The lack of adaptive learning is also demonstrated for the first time in an intra-subject experimental setting, when using as source ten data acquisitions recorded from the same subject but on five different days. This novel result has remarkable repercussions. In fact, in this paper, it was demonstrated for the first time that not only in single-session recordings, but also in an intra-subject experimental setting, adaptive learning is not taking place, and several questions regarding the training of prosthesis with previously acquired data arise. If these results would be confirmed in further studies, the training effort for amputee subjects could not be minimized with the exploiting of the previous knowledge available, at least with algorithms and techniques employed so far. However, authors believe that other strategies (e.g., based on deep neural networks) should be evaluated as well, as they might allow for exploiting prior information thanks to different approaches. These results are in accordance with what was already found when examining the same domain from EMG recordings using other data extraction methods such as muscle synergies [15], in which inter-session analysis was carried out, revealing how data can vary considerably also intra-subject and cannot be used for generalizing intra-subject and inter-subject patterns.

This work has some limitations. First, data from many sessions were used; however, longer time periods could be considered to extend the validity of our results in prolonged recordings. Moreover, while the number of participants is not low for this type of study, it still cannot be considered as representative of all subjects. Future work should expand our results including a higher cohort of volunteers, also divided according to registry data, so that the conclusions could be extended to gender and age differences. Despite our results, we think that classification accuracy of a task may be improved using previous data available from related tasks. Future work needs to consider this problem by conducting experiments with new and different approaches or by using a larger number of acquisitions. Furthermore, it is possible that other classification or pre-processing methods may allow domain adaptation, for instance by taking into account physical constraints (such as physical electrode placement) [38] or by using different transfer learning techniques (e.g., based on deep neural networks).

## 5. Conclusions

Differently from what has been described in several previous studies on domain adaptation in electromyography, our results show that domain adaptation does not appreciably improve classification accuracy, regardless of the transfer learning techniques tested. The results extend previous studies for a realistic setting by using random forests as classification algorithm and two algorithms for domain adaptation.

The lack of adaptive learning is also demonstrated for the first time in an intra-subject experimental setting, when using as source ten data acquisitions recorded from the same subject but on five different days. The results demonstrate that the use of previous experience does not offer concrete improvement, even when considering data from the same subject and a different classifier, confirming and extending previous achievements and somehow posing alternative interpretations with respect to several previous works on domain adaptation. Future works should consider different approaches or use a higher number of repetitions in order to improve the performance of the classifier by employing prior information from related tasks.

## Figures and Tables

**Table 1:** NinaPro Data. Data employed in this study come from DB2, DB3, DB6 NinaPro datasets. The 8 electrode-array was used for the analyses.

DB	N° Sensors	N° Gestures	Repetitions
DB2	Delsys Trigno sEMG × 12	40	6
DB3	Delsys Trigno sEMG × 12	40	6
DB6	Delsys Trigno sEMG × 14(8)	7	12 × 2 × 5

**Table 2:** Classification methods accuracy on datasets DB2 and DB3 for each combination of subjects and for each repetition.

DB2 and DB3													
		**Intact–Intact**				**Amputee–Amputee**				**Intact–Amputee**			
	**Rep**	**1**	**2**	**3**	**4**	**1**	**2**	**3**	**4**	**1**	**2**	**3**	**4**
**Methods**													
Source only		0.150	0.150	0.150	0.150	0.040	0.040	0.040	0.040	0.036	0.036	0.036	0.036
Target only		0.550	0.640	0.680	0.700	0.274	0.335	0.360	0.396	0.270	0.340	0.370	0.390
SER		0.310	0.410	0.470	0.520	0.160	0.210	0.240	0.258	0.180	0.240	0.264	0.290
STRUT		0.540	0.620	0.660	0.680	0.250	0.298	0.320	0.338	0.234	0.290	0.316	0.330
MIX		0.500	0.580	0.630	0.660	0.228	0.278	0.300	0.320	0.234	0.290	0.330	0.350
SER+Target		0.520	0.610	0.660	0.690	0.246	0.304	0.334	0.355	0.256	0.330	0.360	0.381
STRUT+Target		0.560	0.650	0.690	0.700	0.270	0.324	0.350	0.378	0.280	0.350	0.380	0.398
MIX+Target		0.550	0.640	0.670	0.700	0.254	0.312	0.340	0.356	0.274	0.350	0.388	0.398

**Table 3:** Classification methods accuracy on datasets DB6 for each combination of subjects and for each repetition.

DB6							
		**Inter-Subject**					**Intra-Subject**
	**Rep**	**1**	**2**	**3**	**4**	**8**	**8**
**Methods**							
Source only		0.440	0.440	0.440	0.440	0.440	–
Target only		0.588	0.622	0.694	0.710	0.754	0.758
SER		0.500	0.546	0.592	0.620	0.674	0.580
STRUT		0.570	0.618	0.682	0.700	0.728	0.740
MIX		0.600	0.634	0.682	0.700	0.728	0.730
SER+Target		0.620	0.648	0.700	0.714	0.748	0.748
STRUT+Target		0.594	0.630	0.698	0.712	0.754	0.758
MIX+Target		0.610	0.642	0.700	0.714	0.748	0.754

## Data Availability

NinaPro data are available at: http://ninaweb.hevs.ch/, accessed on 1 November 2021.
